# Advances in Cytosolic dsRNA Sensing of Viral Infections

**DOI:** 10.1111/imcb.70163

**Published:** 2026-09-27

**Authors:** Sofia Maceratessi, Natalia G. Sampaio

**Affiliations:** ^1^ Centre for Innate Immunity and Infectious Diseases Hudson Institute of Medical Research Clayton Victoria Australia; ^2^ Department of Molecular and Translational Sciences, School of Clinical Sciences Monash University Clayton Victoria Australia

**Keywords:** autoimmunity, dsRNA, homeostasis, innate immunity, MDA5, OASs, PKR, RIG‐I, viral infection, ZBP1

## Abstract

Detection of abnormal RNA species, such as dsRNA, in the cell cytoplasm underscores innate immune response to viral infections. In humans, the cytosolic dsRNA sensors include RLRs (RIG‐I, MDA5, and LGP2), PKR, and OASs (OAS1, OAS2, OAS3, and OASL). These receptors are all known to bind varying forms of dsRNA and activate differential signaling pathways whose outcome is to limit viral replication. In this article, we describe some of the major recent advances in dsRNA sensing of viral infections, such as the role of endogenous host RNAs in activating sensors, phase separation as a new layer of sensor regulation, cell type‐specific versus universal pathway regulation mechanisms, and the interplay between these pathways within the cell. We have focused on key discoveries of the past 2 years, mainly in mammalian systems.

## Graphical abstract

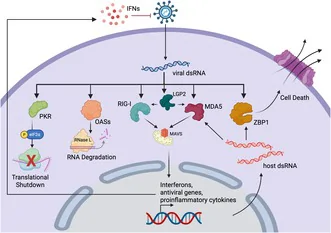

## 1 Introduction

The detection of abnormal RNA species in the cell cytoplasm is a cornerstone of the innate immune response to viral infections. Viruses are obligate intracellular pathogens that must enter and replicate within host cells. In this process, viruses generate abnormal RNAs that are low or absent at homeostasis. Cytosolic RNA sensors detect these abnormal RNAs and induce signaling cascades that lead to antiviral inflammatory responses and/or initiate antiviral programs to inhibit viral replication. This process acts to both repress viral spread and recruit an adaptive immune response to further control infection.

One of the most common forms of abnormal RNA that is generated during viral infection is double‐stranded RNA (dsRNA), which can be formed by complementary base pairing within one RNA molecule or between two separate RNA strands. In humans, the cytosolic dsRNA sensors include the RIG‐I‐like receptors (RLRs; RIG‐I, MDA5, and LGP2), protein kinase R (PKR), and 2′‐5′‐oligoadenylate synthetases (OASs; OAS1, OAS2, OAS3, and OASL in humans). These receptors are all known to bind varying forms of dsRNA but will differ in their pathway outputs (Figure 1). RLR activation results in expression of pro‐inflammatory cytokines, in particular Type I/III interferons (IFNs). PKR phosphorylates the eukaryotic translation initiation factor eIF2α, resulting in global protein translation shutdown. OASs generate the secondary messenger 2′‐5′‐oligoadenylate (2‐5A), which activates the constitutively‐expressed RNase L to degrade total cellular RNA [1]. For some of these sensors, the ligand in the context of a viral infection is well defined, for example, RIG‐I is activated by unmethylated, uncapped RNAs with tri‐ or di‐phosphate groups at the 5′ end. For MDA5, PKR or the OASs, studies have defined approximate dsRNA lengths required for optimal receptor activation, which vary between 30 and 100 bp lengths depending on the receptor [2, 3, 4]. An additional cytoplasmic RNA sensor called Z‐DNA binding protein 1 (ZBP1) detects dsRNA in the Z‐conformation, which constitutes nucleic acids with a left‐handed double helical structure, rather than the canonical right‐handed helix. ZBP‐1 activation initiates cell death signaling pathways, which are cell type and context dependent—for example, necroptosis, pyroptosis, and apoptosis. In this review, we highlight major recent advances in cytosolic dsRNA sensing in the context of viral infection, in particular novel mechanisms of activation and pathway regulation.

**FIGURE 1:**
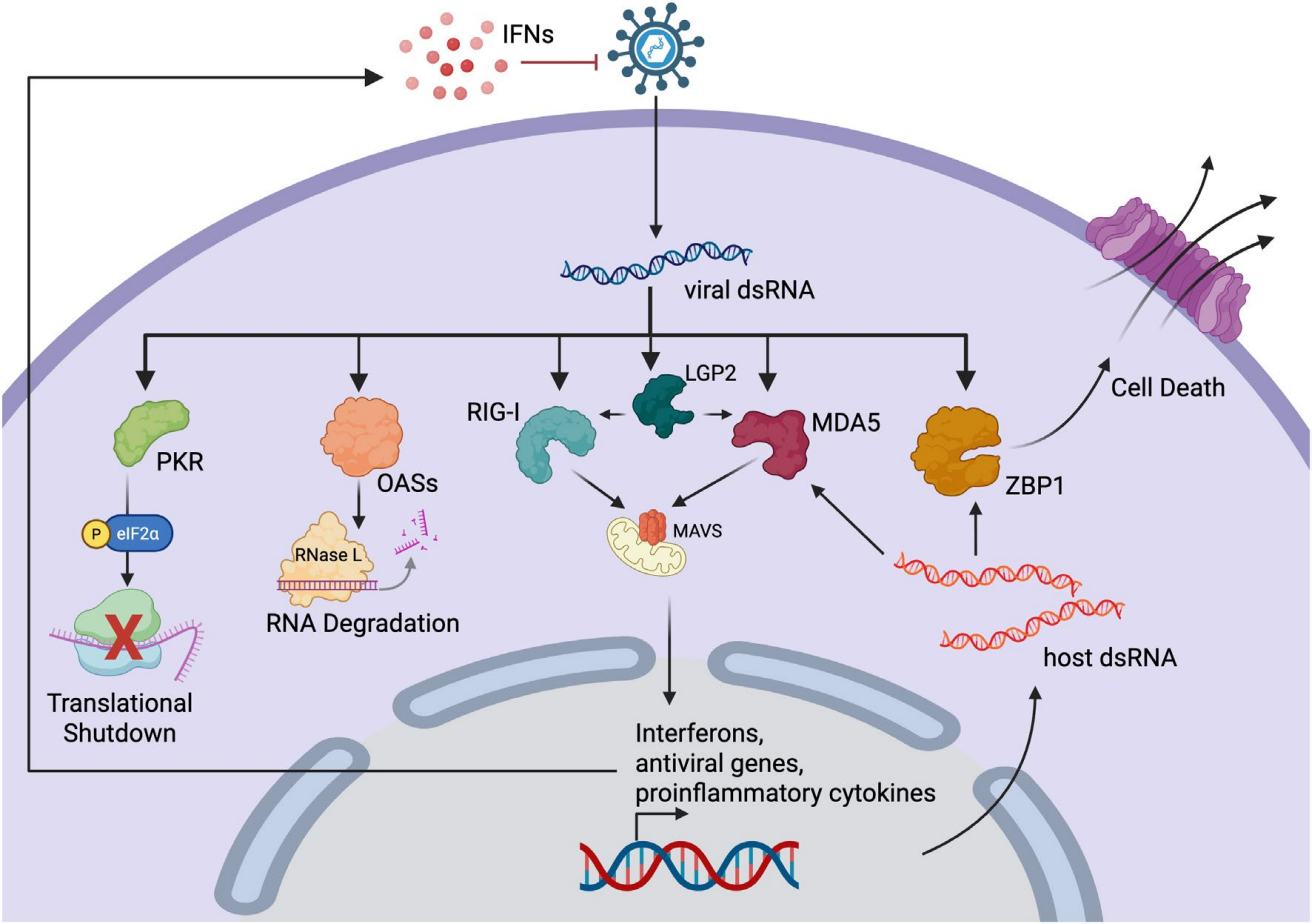
Activation of cytosolic dsRNA sensors by viral infection generates multiple outcomes. dsRNA generated during viral infection, either of viral origin or from endogenous sources, can activate multiple cytosolic dsRNA sensors. Activation of PKR critically induces protein translation shutdown via phosphorylation of eukaryotic translation initiation factor eIF2α. Activation of OASs (OAS1, 2, and 3) results in global cellular RNA degradation via activation of RNase L. Activation of either RIG‐I or MDA5 induces an inflammatory transcriptional response via MAVS at mitochondria, resulting in the expression of cytokines such as interferons and some directly antiviral genes. LPG2 regulates dsRNA binding and activation of MDA5 and RIG‐I. Activation of ZBP1 by dsRNA in the Z‐conformation induces pyroptotic cell death. These mechanisms serve to limit viral spread and signal the host organism to the presence of a viral infection.

## 2 Sensing of Host dsRNA Molecules During Viral Infection

Innate immune receptors, also called pattern recognition receptors (PRRs), have historically been understood to recognize pathogen associated molecular patterns (PAMPs), which are present in pathogen groups but absent in the host—a classic example being the detection of lipopolysaccharides on the surface of gram‐negative bacteria by Toll‐like receptor (TLR) 4 [5]. Later, it was shown that some PRRs sense host‐derived molecules that are mislocalised or are at concentrations outside of homeostasis, such as mitochondrial DNA in the cytosol sensed by the DNA sensor cGAS [6]. In the case of dsRNA sensors, it is believed that the PAMP for these receptors is viral‐derived nucleic acids that are present in the viral particle and/or are generated during viral replication. For example, it has been largely assumed that the dsRNA ligand for MDA5 during infection with positive‐sensed RNA viruses is the viral replicative form, consisting of complementary positive and negative‐sense full‐length viral genomes generated during replication [7]. In the case of RIG‐I, which senses 2′‐O unmethylated duplex RNA containing tri‐ or di‐phosphates at the 5′ end, it has been well established that RIG‐I agonistic RNA is largely absent during homeostasis but generated during specific viral infections, such as Influenza A virus (IAV) infection [8]. However, many of these sensors have also been found to be activated in the absence of viral infection, where they are instead activated by endogenous host RNAs (reviewed in [1]). It was thus largely assumed that activation of dsRNA sensors during infection was driven by viral RNAs, whereas activation during sterile inflammation was driven by endogenous RNAs. This distinction, however, has been challenged by some recent discoveries.

An advancement in the paradigm of RNA sensing during viral infections was made by Yin and Fedorov et al. [9], where they discovered that during infection with herpes simplex virus 1 (HSV‐1) or with IAV, ZBP1 is activated by host‐derived RNA. This was found to be due to the viral infection inducing dysregulation of host RNA transcription, resulting in disruption of transcription termination (DoTT). DoTT was caused by the HSV‐1 protein ICP27 or the IAV protein NS1, both of which disrupted 3′ processing of nascent mRNA, resulting in transcription of RNAs beyond gene boundaries and into regions containing inverted endogenous retroelements. These inverted retroelements have the propensity to form dsRNA structures as well as Z‐form dsRNA. These Z‐form dsRNAs were sufficient to stimulate ZBP1 and consequent cell death. Thus, this work proposes that ZBP1 is a sensor of host RNAs generated as a result of viral infection, rather than a sensor of viral RNAs per se. Whether dsRNAs generated by DoTT can also activate other dsRNA sensors, and if this phenomenon occurs with other viruses, is still to be determined. Nevertheless, it presents a significant shift in our understanding of RNA sensing as a potential guarding mechanism that can be triggered when RNA homeostasis is compromised.

Lork et al. [10] also showed that IAV induced the accumulation of host‐derived inverted retroelements in the cytosol of infected cells, and additionally that this was more apparent in the absence of NS1. They found that NS1 specifically bound to dsRNAs, and posited that NS1 sequesters dsRNAs away from cytosolic RNA sensors. This is in contrast to the finding by Yin and Fedorov et al., who found that NS1 is required to induce the expression of Z‐form dsRNA that activates ZBP1. This discrepancy may arise from differences in cell models, species, and/or experimental methods, as Yin and Fedorov et al. specifically investigated Z‐RNAs in the context of ZBP1 activation, whereas Lork et al. performed a sensor‐agnostic assessment of dsRNA accumulation in cells. Moreover, the IAV NS1 protein has been assigned many roles in host modulation [11], including inhibiting the interferon pathway, which may make it challenging to deconvolute its potential roles in different cellular infection models.

The concept of a dsRNA sensor guarding host RNA homeostasis during infection has recently also been suggested for MDA5 [12]. We reported that during infection with encephalomyocarditis virus (EMCV) and Severe acute respiratory syndrome coronavirus 2 (SARS‐CoV‐2), cellular RNA transcription was imbalanced, resulting in an increase in intronic, upstream, downstream, and divergent transcription of host RNAs in the cytoplasm. During viral infection, MDA5 specifically bound to host RNAs, in particular intronic sequences containing repeat elements like *Alu*. Rescue of aberrant RNAs in the cytoplasm by overexpression of the splicing and RNA export factor SRSF3 prevented aberrant RNA accumulation and MDA5 activation, indicating that host aberrant RNA significantly contributed to MDA5 activation during infection. MDA5 binding to inverted *Alu* repeats had already been proposed as a mechanism of stimulation of gain‐of‐function MDA5 variants that lead to the severe interferonopathy Aicardi‐Goutières syndrome [13]. This new work suggests that sensing of endogenous dsRNA, such as inverted retroelements, may be a major mechanism that activates MDA5, even during viral infections. Thus, how broadly viral infections induce DoTT and other RNA processing imbalances, and whether other dsRNA sensors are similarly activated by these RNAs, is an important open question.

In addition to endogenous retroelements contributing to dsRNA sensor activation, it was recently described that mitochondrial dsRNA may also enhance innate immune responses in a feed‐forward loop. Ku et al. [14] found that SRA stem‐loop‐interacting RNA‐binding protein (SLIRP), a mitochondrial protein that stabilizes mitochondrial RNA (mtRNA) was upregulated in certain autoimmune diseases, like Sjögren's Syndrome. The authors found that both SLIRP and cytosolic mtRNA were increased with RNA sensing, in particular MDA5 activation. They propose that increased SLIRP drives release of double‐stranded mtRNA, which further amplifies antiviral signaling through MDA5 activation. In some viral infections that strongly activate MDA5, such as EMCV, loss of SLIRP decreased interferon responses to infection. This indicates that mtRNA released during some viral infections may be another source of endogenous RNA that activates MDA5.

## 3 Novel Pathway Regulators

Due to their capacity to elicit strong and potentially damaging proinflammatory responses, PRRs have tightly regulated pathways. Regulators of PRRs mostly function by catalyzing post‐translational modifications (PTMs), through direct interactions that promote or inhibit PRR activity, and through regulation of protein turnover. Interactions mediated by distinct RNA molecules have also been described.

Despite the canonical dsRNA sensing event and downstream signaling being generally well defined for dsRNA sensors, in particular for RLRs [8], the regulation and fine‐tuning of these sensors is still being revealed. Identification of critical crossroads of host‐pathogen interaction will provide a better understanding of pathway regulation at the molecular level that may promote the development of antiviral drugs and immune modulatory therapies [15].

### 3.1 Regulation by PTMs

PTMs act as molecular switches that control signaling pathways. By covalently linking functional groups to specific amino acids, PTMs rapidly alter a protein's conformation, stability, subcellular localization, and interactions. Many PRRs are regulated by PTMs that determine the timing and/or magnitude of activation, either to allow rapid and sensitive recognition and elimination of pathogens, or to curb excessive cytokine responses to prevent potentially deleterious outcomes for the host [16].

Serine/threonine phosphorylation, lysine ubiquitination, conjugation of ubiquitin‐like (UBL) proteins, acetylation and deamination are the most well‐characterized PTMs regulating RLR activity. Some of these PTMs blunt RLR signaling: phosphorylation prevents aberrant activation of both RIG‐I and MDA5, K48‐linked polyubiquitylation mediates degradation, and non‐covalent binding of FAT10, a less well‐characterized UBL protein, was reported to sequester signaling‐active RIG‐I from the mitochondria. Meanwhile, other PTMs are necessary for full activation of these signaling pathways: K63‐linked ubiquitin chains contribute to effective RLR oligomerization and signal transduction; SUMOylation of both RIG‐I and MDA5 prevents their K48‐polyubiquitin‐dependent degradation; deamidation of RIG‐I triggers its activation; and finally, reversible acetylation was shown to modulate the ability of RIG‐I to bind to viral RNA [8, 17].

Apart from the well‐known, conventional PTMs, novel PTMs have now been described for RLRs [18]. Palmitoylation was found to be important for stabilizing the aggregation of MAVS, the key downstream mediator for RLR signaling, on the mitochondrial outer membrane [19, 20]. S‐palmitoylation is the covalent attachment of long‐chain fatty acyl groups to proteins on cysteine side chains via thioester bonds. S‐palmitoylation increases the hydrophobicity of the modified domain, typically promoting protein localization to membranes and regulating protein trafficking. Many integral membrane proteins are known to be S‐palmitoylated, but this is the first report of this particular PTM in the context of innate immune signaling. ISGylation, which is the conjugation of the UBL ISG15 to proteins, drastically increases upon activation of innate immune pathways. It had been known that MDA5 is ISGylated [21], but the physiological function of MDA5 ISGylation had not been demonstrated. Sarkar and colleagues [16] showed in multiple viral infection models that MDA5 ISGylation, catalyzed by HERC5 in humans and HERC6 in mice, is crucial for MDA5's ability to form higher‐order oligomeric assemblies and to induce antiviral IFN responses. While traditional modifications (such as phosphorylation) act as well‐established binary switches, these novel PTMs introduce a new contextual layer of regulation that redefines the kinetics and specificity of antiviral signaling pathways. As such, PTMs cease to be isolated molecular events and instead become critical control nodes for host‐pathogen interaction, with the potential to be targeted for more selective and specific therapeutic intervention.

### 3.2 Regulation by Protein Degradation

Ubiquitin, a 76‐amino acid polypeptide with 7 lysine residues (K6, K11, K27, K29, K33, K48, and K63), is conjugated to substrate proteins in multiple forms. Generally, proteins marked with K48‐linked ubiquitin chains are targeted for degradation, whereas their K63‐linked counterparts can promote substrate activation [22]. Ubiquitinated proteins are recognized and degraded through the ubiquitin‐proteasome system or the autophagy‐lysosome pathway, the two primary pathways governing protein turnover in eukaryotes. As with the previously described PTMs, ubiquitination acts as a critical molecular switch in innate immune signaling during viral infections, fine‐tuning the amplitude and duration of antiviral responses by targeting key signaling molecules for proteasomal turnover or orchestrating non‐degradative scaffolding pathways. Numerous RING finger protein family E3 ligases that regulate degradation of PRRs and downstream signaling molecules are continuously being described. Recently, members of the E3 ubiquitin ligase have been associated with innate immune signaling: RNF20 and RNF167 catalyze the ubiquitin‐mediated degradation of RIG‐I and MDA5 [23, 24], RNF144B regulates autophagic degradation of MDA5 [25], and TRIM28 [26] and Ankyrin repeat and IBR domain containing 1 (ANKIB1) promote the degradation of MAVS [27].

Like other PTMs, ubiquitination is reversible and counteracted by deubiquitinating enzymes (DUBs), which hydrolyse ubiquitin chains from target proteins. In the context of innate immune signaling, the work by Tang et al. [28] illustrates this dynamic interplay: the linear ubiquitin chain assembly complex (LUBAC) catalyzes the linear ubiquitination of TRAF6, whereas OTU deubiquitinase with linear linkage specificity (OTULIN) reverses this modification. Notably, the linear ubiquitination of TRAF6, a protein involved in the NF‐kB signaling pathway, is required for the full activation of the antiviral innate immune response and cytokine production. So collectively, the group's findings identified the deubiquitinase OTULIN as a negative regulator of RIG‐I‐dependent IFN‐I signaling.

There is an additional layer of complexity in the ubiquitin regulatory network when considering the proteins that regulate the described E3 ubiquitin ligases/deubiquitinases. This last concept is exemplified by the work of Zeng and colleagues [22] in which they showed that ORMDL sphingolipid biosynthesis regulator 3 (ORMDL3) binds to ubiquitin‐specific protease 10 (USP10), which forms a complex with and stabilizes RIG‐I through decreasing its K48‐linked ubiquitination. ORMDL3 thus disrupts the interaction between USP10 and RIG‐I, thereby promoting RIG‐I degradation and dampening Type I IFN response in the context of viral infection. In a separate work, Wang et al. [29] demonstrated that the exocyst complex protein Secretory 8 (Sec8) stabilizes RIG‐I by inhibiting its ubiquitination and subsequent proteasome‐mediated degradation.

These recent discoveries of ubiquitin‐mediated regulation of the RLR pathway have increased the complexity of our understanding of these receptor pathways [8]. However, how these systems may function in concert is not understood. It is likely that some of these regulators will predominate based on cell type and expression levels. It will be important for future work to consider how these regulators may operate concurrently, or in cell‐ and virus‐specific contexts.

### 3.3 Regulation by Direct Protein Interaction

Many signal transduction pathways are regulated through direct protein–protein interactions. Scaffolding or chaperone proteins can orchestrate the spatial and temporal organization of multiprotein assemblies. This is particularly relevant for the RLRs and downstream adaptor MAVS, which require oligomerization as part of their modes of activation, and for PKR, where homodimerization is an essential step in activation. Recent studies have investigated the role of additional dsRNA binding proteins in regulation of sensor interaction with dsRNA. Previous seminal work showed that MDA5 oligomerization on long dsRNA structures, observed as “filaments” in electron microscopy of in vitro biochemical systems, is critical for MDA5 downstream signaling [4, 30]. MDA5 operates as an ATP‐hydrolysis‐driven motor that translocates along dsRNA in a one‐dimensional manner. Multiple MDA5 monomers can cooperatively load onto a single dsRNA, but their movements rarely synchronize, inhibiting spontaneous filament formation and activation. Two separate studies report how LGP2, (an enigmatic RLR lacking the signal‐transmitting CARDs) facilitates MDA5 activation by impinging MDA5 movement on dsRNA. Using visualization of single MDA5 molecules on dsRNA, Han et al. [31] demonstrated that LGP2 could halt MDA5 molecule “scanning” on dsRNA to promote MDA5 oligomerization. Kurihara and colleagues [32] further identified the filament‐forming interfaces between LGP2 and MDA5 using cryo‐EM and proposed that LGP2 recognizes MDA5 monomers and blocks their movement, thereby promoting filament assembly through a translocation‐directed mechanism. In vitro, LGP2 forms filament‐like assemblies with MDA5 along the internal region of dsRNA, promoting MDA5 filament nucleation that thus stimulates MAVS filament formation and downstream signaling [32]. These works provide important new biochemical evidence of the role of LGP2 in MDA5 signaling, which in the past has been controversial due to contrasting reports on its role as a positive or negative regulator of RLRs during infection [33].

The regulation of dsRNA sensor “scanning” on dsRNA has also been reported for PKR. Recently, Ahmad and colleagues [34] demonstrated that the dsRNA‐binding protein PACT, which was previously reported to support RIG‐I and MDA5 activation [35], can limit PKR scanning on dsRNA, decreasing the rate of homotypic PKR interactions necessary for activation. This increases the threshold for PKR activation to enable tolerance of cellular dsRNA (e.g., endogenous RNA sequences containing *Alu* elements) and preserve homeostasis. The authors showed that PACT's inhibition of PKR involves weak but direct and specific protein–protein interactions between PACT and PKR additional to their independent binding of dsRNA. However, the specific role of PACT modulation of PKR in the context of viral infections remains to be investigated. Collectively, these studies demonstrate a similar underlying mechanism, namely the binding of a regulatory protein (LGP2 or PACT) to dsRNA to prevent sensor scanning, but with divergent functional effects depending on the specific sensor (MDA5 vs. PKR) and cellular context (viral infection vs. sterile conditions).

In addition to modulation of sensor‐dsRNA interactions, protein–protein interactions with other molecules also regulate dsRNA sensor functions. Chaperone proteins facilitate intracellular redistribution and interactions among the signaling molecules. In the case of innate immunity, this process promotes the kinetics of Type I IFN induction and is thus important to restrict viral propagation and infection. Multiple studies have discovered new chaperones in the MDA5 pathway: Annexin A2 (ANXA2) [36], A Disintegrin And Metalloproteinase domain 9 (ADAM9) [37], heat shock protein HSPA6 [38], and 14–3‐3η [15] proteins have been found to regulate the mitochondrial redistribution and/or oligomerization of MDA5 to enable interaction with MAVS, and the subsequent formation of large MAVS aggregates on mitochondrial membranes upon viral sensing. These studies are expanding the signaling network of RLR pathways and provide potential new druggable targets for pathway manipulation. However, as with the multiple E3 ligases recently reported to regulate RLRs, it is likely that cell type and viral infection context will dictate the role of these chaperones in the pathways.

### 3.4 Modulation of dsRNA Sensors by RNA

Although the previously described protein–protein interactions and PTMs are critical for antiviral signaling, there is growing appreciation that proteins and protein complexes can be regulated by interactions with RNA. RNA interactions can allosterically modify protein complex function, or act as chaperones or scaffolds. For example, Rivero and colleagues [39] found that IFN alpha‐inducible protein 27 (IFI27) interacts with MDA5, likely by competing with MDA5 for RNA binding. This interaction could act as a negative feedback loop to dampen excessive inflammation or as a viral mechanism to escape antiviral responses. However, considering the abundance and variety of RNA molecules present in a cell, the function of most RNA‐protein interactions in innate immunity remains unexplored.

Some of these novel interactions are not mediated by canonical RNA‐binding domains like the one described previously, but rather through disordered or uncharacterized domains. An innovative finding by Gokhale et al. [40] showed that MAVS directly interacted with the 3′ untranslated regions (UTRs) of more than 100 cellular mRNAs and that these associations were required for a maximal antiviral response. This novel interaction occurred through a large central domain with conserved disorder and was independent of RIG‐I or MDA5 signaling. This exciting work has opened new lines of investigation into how protein‐RNA interactions may regulate cytosolic innate immune receptors.

### 3.5 Regulation by Transcriptional Control

Control of transcription and alternative splicing provides versatile mechanisms for diversifying immune responses. Viral infections reshape host gene expression programs, giving rise to novel or more abundant isoforms that influence cell fate and immune signaling.

Promoter of Tumor Immune Resistance 1 (PTIR1) is a primate‐specific splice variant of RIG‐I (encoded by the gene *DDX58)* that lacks exon 4. PTIR1 expression, triggered by viral infection or IFN stimulation, suppresses RIG‐I and STAT1 ubiquitination, acting as a negative regulator of innate immunity. Since immune signaling is a double‐edged sword, while protective in inflammatory contexts, PTIR1 restricts antiviral defense by dampening IFN responses during viral infection [41].

Evidence of regulation of innate immune pathways at the gene expression level has been provided by Zeng and colleagues [42]. They demonstrated that heterogeneous nuclear protein U (hnRNPU) plays a pivotal role in innate immunity by facilitating chromatin opening to activate expression of OAS3, which mediates an RNase L‐dependent mechanism to enhance innate immune responses against Hepatitis B virus. RNF20, the E3 ubiquitin ligase that catalyzes the ubiquitin‐mediated degradation of RIG‐I and MDA5 mentioned in a previous section, resides in the nucleus, where it maintains immune readiness by regulating the basal and inducible transcription of RIG‐I and MDA5. This demonstrates that some regulators may contribute to immune homeostasis through several mechanisms.

### 3.6 Regulation Beyond Canonical Cascades

New studies have begun to explore the crosstalk between dsRNA sensors and other independent signaling pathways activated by distinct cellular or environmental stimuli. Liu and colleagues [38] discovered that HSPA6, a stress‐induced member of the heat shock protein (HSP) family, is upregulated by interferon‐competent RLRs. Interestingly, HSPA6 is not a canonical ISG, and its upregulation occurs independently of interferon regulatory factor 3 (IRF3) or NF‐κB, but rather through pathways activated by E3 ligases associated with RLRs. A role for hypoxic inducible factor (HIF) signaling pathway in limiting pneumoviral infections was shown by Ha et al. [43], demonstrating that stimulation of this pathway enhanced innate immune signaling and limited viral replication study. Finally, Harrison et al. [44] described the noncanonical UBXN9‐GLUT4 axis can control antiviral immunity via plasma membrane tethering of cytosolic RLRs, discussed in more detail later in this review. These findings uncover previously unknown feedback axes in cell signaling and raise the interesting question of whether other multimeric events can regulate responses to dsRNA stimuli beyond the canonical cascades.

## 4 Subcellular Localisation as a Regulatory Axis in dsRNA Sensing

There is a growing body of literature describing subcellular localization as an important regulatory mechanism in innate immune signaling. Host cellular networks like the cytoskeleton, membrane‐bound organelles, and membraneless biomolecular condensations play roles in innate immune signaling by facilitating the higher‐order assembly of PRRs and regulators.

Some viruses, like Vesicular Stomatitis Virus or members of the Herpesviridae family, can interact with endosomes during viral entry, and it has been demonstrated that the adaptor TBK1‐associated protein in endolysosomes (TAPE) plays a role in regulating the endosomal TLR3 as well as cytoplasmic RLR pathways [45, 46]. Recently, Chen et al. [18] have revealed the spatiotemporal interplay among RIG‐I, TAPE, and endosomes in cells following RNA ligand and virus challenges. The authors report that endosomes act as platforms for facilitating RIG‐I complex formation and that TAPE mediates the interaction of RIG‐I and TRIM25, enhancing RIG‐I activation by promoting its polyubiquitination. Whether other PRRs interact with endosomes or other organelles for activation warrants further investigation. Given TAPE's role in endosomal trafficking, investigating other cellular housekeeping proteins recruited by innate immune pathways during viral infection is an intriguing new direction. Just as viruses hijack host machinery for replication and survival, cells may similarly repurpose housekeeping proteins from their canonical functions to mount a protective defense.

Following on this idea, activation of PRRs can lead to metabolic reprogramming, where metabolites feedback to fine‐tune immune responses. This rewiring of cellular metabolism hinges on glucose transporters (GLUTs). The relationship between GLUTs and immune function has been best described for GLUT1, which facilitates extensive glycolytic reprogramming in innate immune cells following TLR activation. However, the role of other GLUTs in innate immunity was entirely unknown. Harrison et al. [44] uncovered a whole new regulatory axis: at steady state, GLUT4 is trapped at the Golgi matrix by ubiquitin regulatory X domain 9 (UBXN9); following EMCV infection, GLUT4 is released and translocated to the cell surface where it spatially segregates a significant pool of cytosolic RLRs, inhibiting IFN‐β responses.

That spatial segregation of cytosolic molecules, a physical process where a uniform mix of molecules splits into a distinct dense droplet phase, has been termed liquid–liquid phase separation (LLPS; Figure 2A) [47]. Previously a niche topic, LLPS, also described as biomolecular condensation, is now carving out a prominent space in the study of cytosolic PRRs [47]. Shi and colleagues [48] showed that the host factor zinc finger CCHC domain‐containing protein 3 (ZCCHC3) positively regulates both RLRs‐ and cGAS‐mediated signaling through this mechanism (Figure 2B). Rather than developing strong protein–protein interactions, ZCCHC3 harbors multiple nucleic‐acid‐binding modules and undergoes robust liquid phase condensation with nucleic acids. RNA‐induced ZCCHC3 condensates spatially enrich and activate RLRs, which facilitates the interaction with downstream factors. In contrast, Hou and colleagues [49] found that Toll‐interacting protein (TOLLIP) forms condensates that promote deSUMOylation and reduced aggregation of MAVS, dampening IFN responses (Figure 2C). Moreover, Zheng et al. [50] have demonstrated that the RNA‐binding protein Cold Shock Domain Containing E1 (CSDE1) forms RNA‐dependent biomolecular condensates that sequester immunostimulatory viral RNAs and shield them from RLR recognition in macrophages (Figure 2D). Therefore, there is a contrasting duality in this mechanism of aggregation of nucleic acids and proteins. While in some cases it may facilitate interaction between ligands, receptors, and pathway regulators, in other cases it can shield nucleic acid recognition or promote the binding of negative pathway regulators. Again, it is likely that differences in cell type and infection will be relevant to the dynamics of LLPS in dsRNA sensing, and that the function of LLPS will vary temporally over the course of infection and pathway activation.

**FIGURE 2:**
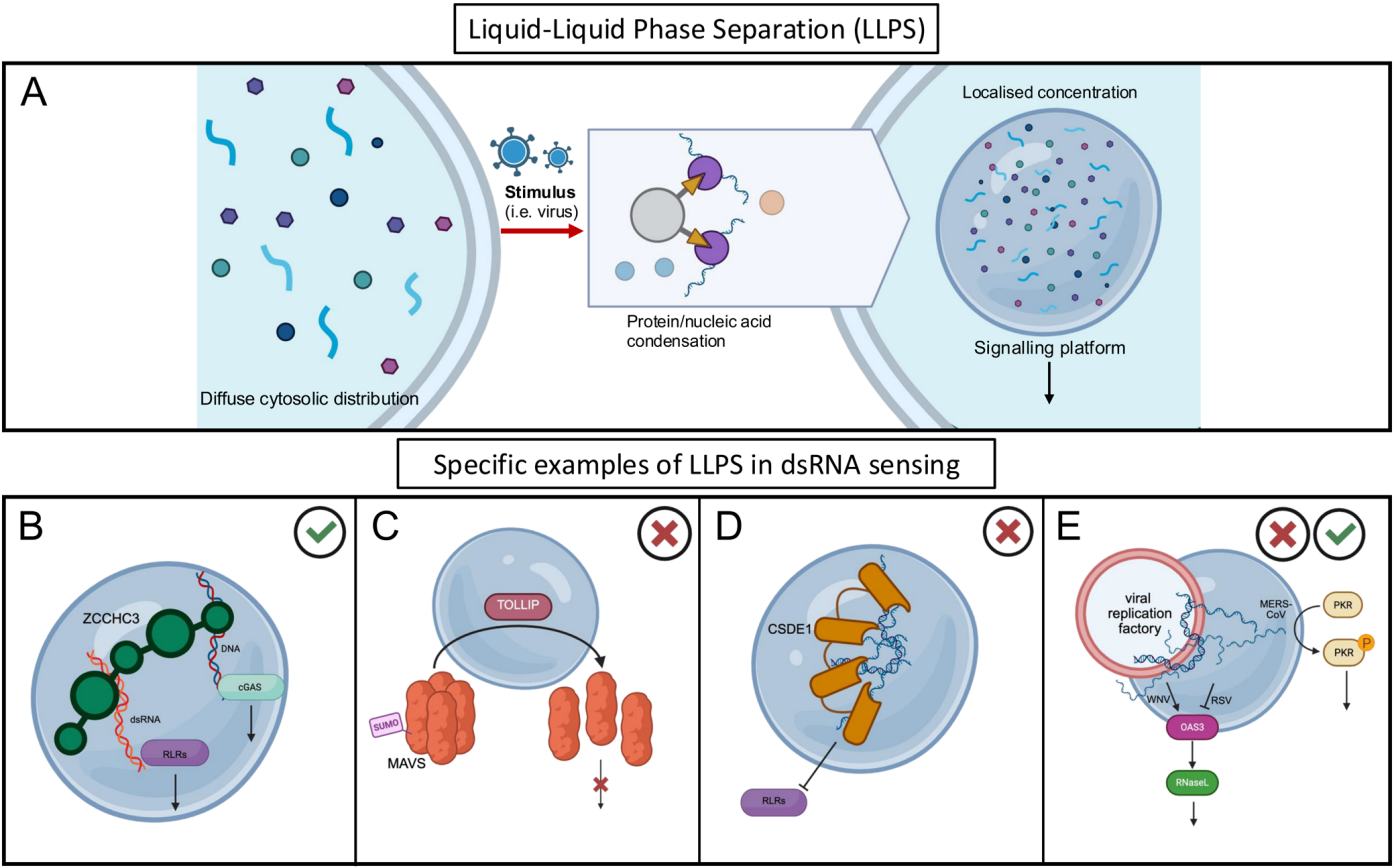
Liquid–liquid phase separation (LLPS) in dsRNA sensing. (A) LLPS is a physical process where a uniform mix of molecules aggregates into a distinct dense droplet phase. This process enables localized concentration of molecules, which can be proteins and nucleic acids, to modulate signaling pathways. Specific examples of LLPS or protein condensation in dsRNA sensing include: (B) ZCCHC3 binding to nucleic acids to enrich ligand concentration and promote RLR and cGAS activation; (C) TOLLIP condensation promotes deSUMOylation of MAVS to reduce IFN responses; and (D) CDSE1 binds and sequesters RNA away from RLRs to prevent their activation. (E) In some instances, condensation can prevent RNA access to OAS3 (e.g., Respiratory syncytial virus RSV) whereas in other infections this promotes OAS3 activation (e.g., West Nile virus WNV). Condensation of PKR near viral replication factories promotes it is robust activation in Middle Eastern respiratory syndrome coronavirus (MERS‐CoV) infection.

OAS3 condensation on dsRNA proximal to West Nile virus replication complexes was recently described as necessary to initiate RNase L activation [51]. On the other hand, Hwang et al. [52] have shown that Respiratory Syncytial virus forms phase‐separated biomolecular condensates that serve as hubs for viral replication, which actually protect viral RNA from the OAS‐RNase L pathway (Figure 2E). Why some protein aggregates facilitate OAS‐RNase L activation and others shield nucleic acid from recognition remains to be explained. In terms of studying the spatiotemporal and stoichiometric regulation of this process, Cusic and Burke [53] employed several imaging techniques to study RNase L condensation in response to flavivirus infection. They describe the formation of granules composed of an OAS3‐dsRNA core surrounded by a shell of RNase L, which dynamically exchanged with a cytoplasmic pool. Here, condensation enabled rapid activation of RNase L, and may also set a threshold to prevent spurious activation. Blomqvist and colleagues [54] also employed imaging techniques to study this phenomenon in PKR activation. During infection with Middle Eastern respiratory syndrome coronavirus, PKR activates upon condensation on dsRNA exposed at membrane‐associated viral replication complexes (Figure 2E). Subsequently, phosphorylated PKR (p‐PKR) condensates dissociated from dsRNA and dissolved, releasing activated PKR molecules into the cytosol for downstream signaling. Importantly, the disassociation of p‐PKR from dsRNA allowed for the exchange of inactive PKR monomers, thus promoting robust PKR activation from limited exposed viral dsRNA substrates [54].

This LLPS process can be further pushed, transforming into liquid–solid phase transition (LSPT) a state where cellular components shift between functional fluid droplets to form nonfunctional rigid aggregates. This is evidenced by a report on inborn errors of the human RNA lariat–debranching enzyme 1 (DBR1), which causes an accumulation of RNA lariats. Lariat RNAs are splicing intermediates produced by Group II introns and the pre‐mRNA spliceosome. Intriguingly, although patients with DBR1 deficiency likely accumulate RNA lariats in multiple cell types, they have a narrow but devastating susceptibility to viral brainstem encephalitis. Ru and collaborators [55] demonstrated that the accumulation of RNA lariats in human DBR1‐deficient cells interferes with stress granule (SG) assembly, promoting the proteasome degradation of key components of SGs. Unlike linear RNAs, RNA lariats bind and form puncta with Ras GTPase‐activating protein‐binding protein (G3BP) separately from SGs. They therefore interfere with the LLPS of G3BPs and generate G3BP aggregates that lack liquidity, represent a solid state, and disengage other SG components. The abnormal G3BP condensates are prone to ubiquitin‐mediated degradation. This SG disaggregation in turn impairs PKR activation and activity against viruses, including HSV‐1. PKR can be activated by dsRNA directly, but SGs operate as a platform providing optimal PKR‐eIF2α cascade activation and restriction of viral replication. The disruption of this signaling microenvironment drastically affects PKR activity. Interestingly, this study highlights the critical role that PKR may play in the protection of the brain from viral infection.

As expected, viruses can hijack phase separation to remodel host complexes into pathological aggregates. Spring viremia of carp virus phosphoprotein (SVCV‐P) hijacks the TBK1‐IRF3 signaling axis via liquid–solid phase transition (LSPT), sequestering IRF3, and inhibiting IFN production [56]. In detail, upon stimulation, IRF3 facilitates TBK1 into functional LLPS condensates, spatially enhancing IRF3 phosphorylation, and downstream IFN responses. IRF3 acts as a scaffold upon which TBK1 can be incorporated. Conversely, SVCV‐P undergoes robust LLPS, competitively recruiting TBK1 into SVCV‐P‐TBK1 condensates. These condensates merge with IRF3‐TBK1 droplets, forming SVCV‐P‐TBK1‐IRF3 ternary condensates. These subsequently undergo LSPT, immobilizing IRF3, and preventing its nuclear translocation.

These new in‐depth insights into the mechanisms of PRR activation enable the development of methodologies and therapeutics that hold promising implications in the clinic, as exemplified by Wang et al. [57]. An intermolecular C864‐C869 disulfide linkage, a critical step for human RIG‐I activation, mediates conjugation of an SDS‐resistant RIG‐I oligomer, which prevents RIG‐I degradation by E3 ubiquitin‐ligase MIB2 and is necessary for RIG‐I to perform liquid–liquid phase separation to compartmentalize downstream signalosome. Using unnatural amino acids to generate covalent C864‐C869 linkage, and the development of an interfering peptide to block C864‐C869 residues, the authors bidirectionally regulated RIG‐I activities in human diseases. Because IFN production is essential for antiviral protection yet drives inflammatory damage when excessive, the ability to augment or reduce its activation holds great potential for treating both viral infection and autoimmune diseases.

## 5 Cell‐Type Specific Regulation of dsRNA Sensing

The relative importance of a PRR may be entirely dependent on its cellular context. A receptor that is indispensable for orchestrating the defense against a specific pathogen in one cell type may play a minor or redundant role in another. Therefore, the architecture of innate immunity should not be viewed as a static, generalized network, but rather as a highly specialized, compartmentalized system where the importance of a PRR can be defined by the cell type expressing it and the specific pathogen it encounters.

The highly localized nature of certain novel immune pathway regulators suggests that these interactions are context‐dependent, restricted to specific tissues or cell types rather than applicable to all cell populations. This is particularly relevant in the context of viral infection given that many viruses have selective cellular tropism. For example, in keratinocytes, the Cathelicidin peptide, LL37 has been proven to increase Type I IFN responses [58] upon stimulation with intracellular dsRNA, whereas the stress‐induced keratin 16 intermediate filament gene (KRT16) negatively regulates Type I IFN signaling and innate immune responses in skin after treatment with synthetic dsRNA poly(I:C) [59]. A cardiomyocyte‐enriched structural protein, Junctophilin‐2 has been attributed a novel role in suppressing the automatic activation of innate immune responses in the heart following poly(I:C) treatment [60]. Moreover, the insulin‐responsive GLUT4, which we described earlier as a novel inhibitor of RLR signaling, would operate largely in adipose and muscle tissues where it is expressed [44]. Thus, mechanisms of dsRNA sensor regulation can be not only viral agent‐, but also tissue‐ and cell type‐specific. It is important to highlight that most of these previously mentioned studies did not focus on a specific viral agent, but instead used poly(I:C) treatment to mimic the accumulation of cytosolic dsRNA following viral infections. It will be important for future studies to evaluate whether the mentioned cell‐specific regulatory mechanisms apply in the context of specific viral infections.

## 6 Intercellular dsRNA Sensor Communication

Historically, innate immune sensing pathways have been viewed strictly as cell‐intrinsic. A big breakthrough in the field was the discovery of immune‐specific secondary messengers acting as extracellular/intercellular transmitters. A landmark paper by Ablasser et al. originally established that cGAMP, a secondary messenger generated by the DNA sensor cGAS, does not stay confined to an infected cell and instead passes directly from a dying or infected cell into adjacent healthy cells via Connexin gap junctions [61]. This allows the bystander cell to activate the downstream signaling mediator STING and upregulate Type I IFNs before it ever encounters free DNA or virus. More recent discoveries on this particular form of short‐range communication, which parallels classical neurotransmission or paracrine signaling, have been recently reviewed by Blest & Chauveau [62]. Now, Huai and colleagues [63] have shown that the small linear nucleotide second messenger 2‐5A of the OAS‐RNase L pathway can also be transferred from cell to cell via gap junctions, remotely activating RNase L in recipient cells ahead of the viral replication wave before they are even infected, suggesting that 2‐5A is an immunotransmitter similar to cGAMP.

Immunotransmitters represent a new mode of rapid, short‐range communication between cells. Unlike cytokines and chemokines, which require specific receptors on the target cell, 2‐5A and cGAMP rely on widespread gap junctions and transporters that also transfer other molecules. Their intracellular targets, RNase L and STING, are also broadly expressed. This limited specificity in cell‐to‐cell transfer of immunotransmitters could provide an advantage during the early phase of an infection. Furthermore, immunotransmitters are produced almost immediately after viral nucleic acids bind to the OAS and cGAS sensors, probably within minutes. In comparison, it typically takes hours for the same innate immune signaling pathway to induce cytokine and chemokine production (which involves transcription and translation).

Another novel frontier in dsRNA sensing that has been opened is the intercellular movement of RNA molecules. So far, it has been scarcely reported but it has the potential to become a subject of paramount interest within the discipline. While studying interspecies chimerism, Hu et al. [64] unexpectedly identified widespread bidirectional RNA transfer between co‐cultured mouse and human cells, occurring in a non‐selective, contact‐dependent manner, with MAVS‐dependent RNA sensing playing a critical role. Collectively, these observations have broader implications for understanding intercellular communication and immune surveillance during early embryonic development and across evolutionary boundaries. Similarly, in plant biology, the work by Brosnan and collaborators [65] challenges the dogma around the stability, uptake and movement of dsRNA by demonstrating that dsRNA moves and is subsequently maintained as a largely unprocessed molecule in multiple distal tissues in a variety of model plant and crop species. The authors prove that mobile dsRNA is functional against fungal pathogens in distal tissue types of whole plants. Functionality occurs via dsRNA being transferred from the plant to the fungus, upon which it is processed via the fungal RNAi machinery to elicit gene silencing. It is tempting to speculate whether analogous phenomena occur within animal systems. Ultimately, as these novel mechanisms come to light, the emerging paradigms have the potential to redefine our current understanding of innate immune signaling and cellular responses to viral infections.

## 7 Functional Duality in the OAS‐RNase L Pathway

The OAS‐RNase L pathway is one of the oldest innate RNA sensing pathways. Degradation of cellular mRNA by RNase L is a conserved response to flaviviruses, including Zika virus, dengue virus serotype 2, and West Nile virus [66]. However, how cells balance translational shutdown to limit viral replication with the transcriptional induction of antiviral components like IFNs is not fully clarified. RNase L broadly downregulates the homeostatic cellular proteome, reducing proteins with short half‐lives involved in cell cycle progression, cellular metabolism, and protein synthesis. However, the mRNAs encoded by ISGs can evade mRNA decay by RNase L, allowing for expression of ISG‐encoding mRNAs [67]. Harioudh and colleagues [68] further studied this phenomenon and described a mechanism distinct from canonical RNase L activation in which OAS1 binds AU‐rich elements of specific mRNAs, including IFNβ. This binding leads to the sequestration of IFNβ mRNA to endomembrane regions, resulting in prolonged half‐life and continued translation. In parallel, the same group also described a critical antibacterial role for this classic antiviral effector [69].

A long‐standing conundrum of the OAS‐RNase L pathway is its powerful enzymatic amplification of RIG‐I‐compatible 3'RNA ends (through RNase L cleavage of cellular and viral RNA) and yet its modest contribution to the overall IFN production during infection. In their work, Yang et al. [67] show that the human SKI complex, a cofactor complex that recruits RNA substrates for degradation by the cytoplasmic RNA exosome, plays a crucial role in limiting the antiviral activities of the OAS‐RNase L pathway. Ultimately, these findings reveal that the OAS‐RNase L axis is not merely a blunt antiviral switch, but a highly coordinated arbiter of host RNA homeostasis. This intricate duality, where cellular decay coexists with the privileged preservation and spatial sequestration of critical transcripts, underscores a multi‐layered regulatory architecture that remains an unresolved frontier in innate immunology.

Bell and colleagues [70] have written another intriguing chapter in the arms race between viruses and their hosts. During SARS‐CoV‐2 infection, the key protective OAS that activates RNase L upon sensing viral dsRNA is the membrane‐anchored OAS1 isoform p46. OAS1 p46 is unique among OAS1 isoforms in undergoing prenylation at Cys397 [71, 72]. Prenylation is an irreversible PTM in which hydrophobic isoprenoid lipids are covalently attached to conserved cysteine residues at the C‐terminus of target proteins. This lipid anchoring is essential for targeting proteins to cell membranes, and in the particular case of OAS1, extending the sensing of viral dsRNA to endomembrane niches where coronaviruses replicate, thereby conferring a distinct antiviral advantage beyond the alternate cytosolic OAS1 p42 isoform. Consistent with this, the cleaved form of p46 is redistributed to the cytosol, reducing the sensing of SARS‐CoV‐2 dsRNA and thereby weakening canonical RNase L activation. Bell et al. [70] show that SARS‐CoV‐2 3C‐like main protease (3CLpro/Mpro) efficiently cleaves OAS1 p46, excising the prenylated‐Cys397 membrane anchor, which releases OAS1 p46 from endomembranes. This dampens the cell's antiviral restriction. However, 3CLpro also cleaves RNase L, which removes an inhibitory N‐terminal domain and generates a proteolytically activated RNase L that can act as an antiviral effector. As the authors explain, the conserved RNase L 3CLpro cleavage motif functions as an automatic emergency safeguard that preserves antiviral restriction despite previous OAS1 p46 evasion. This is the first report of proteolytic activation of RNase L by viral proteases. Here the authors not only reinforce the resilience of the OAS‐RNase L axis but also reveal an intrinsic evolutionary constraint on immune evasion. Whether other viral proteases similarly trigger RNase L warrants further investigation.

## 8 Open Questions and Concluding Remarks

Recent advances in dsRNA sensing of viral infections, such as activation by endogenous host RNAs, the role of LLPS in sensor regulation, and the cell type‐specific rules of sensor regulation, represent important areas of active investigation. However, most processes have been studied using isolated individual sensors, and often in single infection models or using dsRNA mimics, leaving it unclear whether these mechanisms apply broadly. Furthermore, despite significant ligand overlap among sensors like MDA5, PKR, and OASs, these pathways are rarely co‐evaluated. Because these receptors are often co‐expressed or co‐induced by interferons during infection, whether they collaborate or compete for the same dsRNA molecules remains a major knowledge gap. For instance, while endogenous host RNAs activate both ZBP1 and MDA5 during viral infections, and shared regulators like PACT act on both PKR and MDA5, inter‐receptor crosstalk and regulation are rarely addressed. Elucidating these simultaneous interactions will be essential to map out the coordinated innate immune response to viral infections.

Additionally, it is important to understand how dsRNA sensor regulation occurs within specific cellular and pathogen contexts. As highlighted throughout this review, pathway regulation varies considerably depending on cell type and infectious agent. Future work investigating new regulatory mechanisms in primary or infection‐relevant cells is critical. Lastly, while in vitro techniques have been essential for unmasking novel molecular mechanisms (e.g., protein oligomerization and RNA‐protein interactions), translating these findings into physiological in vivo models will be essential to understand their functional impact on disease.

## Author Contributions

**Natalia G. Sampaio:** conceptualization, writing – original draft, writing – review and editing. **Sofia Maceratessi:** conceptualization, writing – review and editing, writing – original draft.

## Conflicts of Interest

The authors declare no conflicts of interest.

## Data Availability

Data sharing not applicable to this article as no datasets were generated or analysed during the current study.
